# Humoral immune response to MUC5AC in patients with colorectal polyps and colorectal carcinoma

**DOI:** 10.1186/1471-230X-6-4

**Published:** 2006-01-12

**Authors:** Belma Kocer, John McKolanis, Atilla Soran

**Affiliations:** 1 Research Fellow, Department of Immunology, University of Pittsburgh School of Medicine, Pittsburgh, PA, USA; 2 Senior Research Associate Department of Immunology, University of Pittsburgh School of Medicine, Pittsburgh, PA 15261, USA; 3 Professor of Surgery, Magee-Womens Hospital of UPMC, 300 Halket St, Suite 2601, Pittsburgh, PA 15213, USA

## Abstract

**Background:**

MUC5AC is a secreted mucin aberrantly expressed by colorectal polyps and carcinoma. It has been hypothesized that aberrant expression of MUC5AC in colorectal carcinoma tissues increased the overall survival of patients with colorectal carcinoma. The present study investigates the incidence of naturally occurring MUC5AC antibodies in the sera of normal individuals, patients with colonic polyps and patients with advanced colorectal carcinoma. A second aim was to determine the relationship of MUC5AC antibody with the prognosis of colorectal carcinoma.

**Methods:**

Free circulating MUC5AC antibodies were measured using an enzyme-linked immunosorbent assay with a synthetic peptide corresponding to an 8 aa. segment of MUC5AC tandem repeat region. Immunohistochemical analysis was completed to demonstrate MUC5AC expression in the polyp specimens.

**Results:**

MUC5AC antibodies were detected in 6 of 22 (27.3%) healthy subjects, 9 of 20 (45%) polyp patients, 18 of 30 (60%) patients with colorectal cancer. The presence of circulating free MUC5AC antibody levels was significantly correlated with expression of MUC5AC in polyp sections. Serum MUC5AC antibody positivity was higher in patients with colon located tumors, advanced stage and poorly differentiated tumors were found negatively affecting patient survival in our study. MUC5AC antibody positivity was higher in patients with poor prognostic parameters. Disease free survival and overall survival were shorter in this group of patients. In the multivariate analysis MUC5AC antibody positivity didn't find an independent prognostic factor on prognosis.

**Conclusion:**

Decreased survival in colorectal carcinoma patients with MUC5AC antibody positivity may be due to a decrease in the MUC5AC expression in tumor tissues of surviving carcinoma patients.

## Background

Mucins are high molecular weight glycoproteins with O-linked oligosaccharides attached to serine or threonine residues of the apomucin protein backbone [1]. To date, 19 genes coding for apomucin have been identified [2-5]. Mucins are expressed with a cell and tissue-specific pattern in normal tissues [6,7]. There are two structurally and functionally distinct classes of mucins; secreted gel-forming mucins and transmembrane mucins. Secreted gel-forming mucins include the products of the MUC2, MUC5AC, MUC5B and MUC6 genes on chromosome 11p15.5 [8-10]. Each has a central region with a variable number of tandem repeat (VNRT), but there is a little similarity. MUC5AC was cloned from tracheobronchial [11] and stomach [12] cDNA libraries. Tandem repeat units have eight amino acid residues [12].

MUC5AC expression is found on apical epithelial cells of the mucus glands of gastric antrum and body, tracheobronchial epithelium, superficial epithelium of the gallbladder and endocervix epithelium [7,13-22]. MUC5AC is found in fetal [23,24] and precancerous [25] colonic mucosa but <20% of normal colon tissue [7,25-28]. De novo expression was shown in >55% of colonic polyps. MUC5AC is highly expressed in adenoma. Levels decrease with increasing degree of dysplasia in polyps [25,26,29,30]. Less than 30% of colorectal carcinomas expressed MUC5AC [23,30]. However, in another study, there was de novo expression of MUC5AC in 23/36 colorectal carcinomas [31]. In our previous study, we reported 34.1% of colorectal carcinomas expressed MUC5AC [32]. We emphasized that MUC5AC expression decreases with increased malignancy pathology and MUC5AC negative tumors had a more malignant potential, as shown by a more aggressive behavior. These patients had a significantly shorter survival in our study. We suggested that the absence of MUC5AC expression in colorectal carcinomas might be a negative prognostic factor.

Humoral and cellular immune responses to other mucin core proteins have been described in cancer patients. Tumor reactive cytotoxic T-lymphocytes specific for MUC-1 core peptides have been described in breast [33], pancreatic [34] and ovarian [35] cancer patients. Circulating immune complexes [36,37] and free anti-mucin antibody [38-40] against the MUC 1 tandem repeat also have been identified in patients with benign and malignant tumors.

The specific aims of the present study are to investigate the incidence of humoral immune response against MUC5AC core protein in healthy individuals, patients with colorectal polyps and colorectal carcinoma, and the possible clinical importance of this antigen for the diagnosis and prognosis of colorectal carcinoma.

## Methods

Serum samples were obtained from 22 healthy donors, from 20 patients with colonic polyps, and from 30 colorectal carcinoma patients with recurrent or progressive disease treated in the University of Pittsburgh Medical Center. Serum samples were collected, and stored at – 70 degrees until analyzed. Tissue sample from polyp patients were obtained from the Pathology Department. Colorectal carcinoma tissues were not available for this study.

### Healthy subjects

Mean age of healthy donors was 47.6 years (range 30–80 years) and half of them were male.

### Polyp patients

Mean age of polyp patients was 58.6 years (range 34–74). Forty-five percent of them (n = 9) were female and 55% of them (n = 11) were male. A total of 43 polyps were analyzed; 21% of them were hyperplastic, 67.5% were tubular adenomatous, 9.2% were tubulovillous and 2.3% were villous type. Polyp size was classified as < 1 cm, 1–2 cm, > 2 cm. Seventy five percent of polyps were smaller than 1 cm, 5.6% were between 1–2 cm and 1.4% of polyps were bigger than 2 cm size. Only 4.2% of polyps showed dysplastic changes.

The patients were divided into three groups in order to show relationship between antibody level and having of polyps at past or present time. Group I (55%) patients were newly diagnosed, group II (30%); had a present and past history of colonic polyps, group III (15%) had a past history of polyps only.

### Colorectal cancer patients

Median age was 59 years (range 22–71). Clinicopathologic parameters of these patients were given in Table 1; Tumors were staged according to the modified Dukes' classification proposed by Astler and Coller [41]. Histologic typing of tumors was performed using the World Health Organization classification [42]. The duration of follow-up ranged from 7–92 months (median 39 months). All of the patients died during the follow-up period.

### Synthesis of tandem repeat peptides

MUC5AC peptide sequence TTSTTSAP was prepared by the Peptide Synthesis Facility at the University of Pittsburgh.

### Elisa for detection of mucin-specific antibodies

Circulating free antibodies to MUC5AC were measured with an Enzyme-linked immunoassay (ELISA). The peptide was dissolved in Phosphate Buffered Saline (PBS) at concentration 5 μg/ml. The solution (100 μl) was dispensed into 96-well plates and incubated overnight at 4°C. The plate was washed 3 times with PBS and the remaining protein-binding sites blocked by a 1 hour incubation with 100 μl of 2.5% BSA (Bovine serum albumin) (Sigma, St Louis, MO) in PBS. The control plate has no MUC5AC peptide. Serial plasma dilutions of 1:20-1:160 were prepared in 2.5% BSA. After 1-hour incubation with 50 μl of primary antibody, plates were washed 5 times with 0.1% tween 20 in PBS. The plate was then incubated for 1 h with 50 μl of the secondary antibody consisting of goat antihuman polyvalent (IgM, IgA, IgG) antibody (Sigma Chemical Co. St. Louis, MO) diluted 1: 1000 with 2.5% BSA. The plate was washed 5 times and 100 μl substrate was added. Sigma 104 Phosphatase Substrate was used at 3 mg/ml in 0.05 M NaCO3, 0.5 mM MgCl2. The reaction was stopped after 1 hour of incubation in the dark with 50 μl of 0.5 M NaOH and the Optical Density (OD) read at 405 nm. Each result from the MUC5AC + plate is subtracted from the MUC5AC – control plate, results are a mean of three replicates.

The isotype determination of antimucin antibody was carried out as described above using IgG or IgM specific secondary antibodies (Sigma, St. Louis, MO).

### Immunohistochemistry

Monoclonal anti-human gastric mucin antibody (clone 45M1; Sigma, St. Louis, MO) was used to determine MUC5AC expression in polyp tissues. This antibody recognized the mucin epitope "g", located in the peptide core of MUC5AC [29]. Endocervical was used as a positive control [32]. Anti-MUC5AC antibody was diluted 1:200 with BSA. Sections were stained by immunoperoxidase technique using the Vectastain Elite ABC kit protocol (Vector Laboratories Inc., Burlingame, CA). Antibody expression higher than 10% of the tumor recorded as positive expression in immunohistochemical study [32].

### Statistical analysis

Statistical analysis was performed using the program SPSS advanced statistics 10.0 software package (SSPS Inc, Chicago, Illinois, USA). Differences of MUC5AC serum antibody levels between groups were analyzed with Mann Whitney U test. Differences between the MUC5AC antibody positivity within groups were evaluated with Chi-square test, relationships between clinicopathologic parameters and MUC5AC antibody positivity were evaluated with Chi square or, when the sample size was small, a Fischer's exact test. The probability of parameters on Disease Free Survival (DFS) and Overall Survival (OVS) was analyzed using Kaplan-Meier method. Multivariate analysis of parameters on DFS and OVS was performed using Cox regression models.

## Results

**Figure 1:**
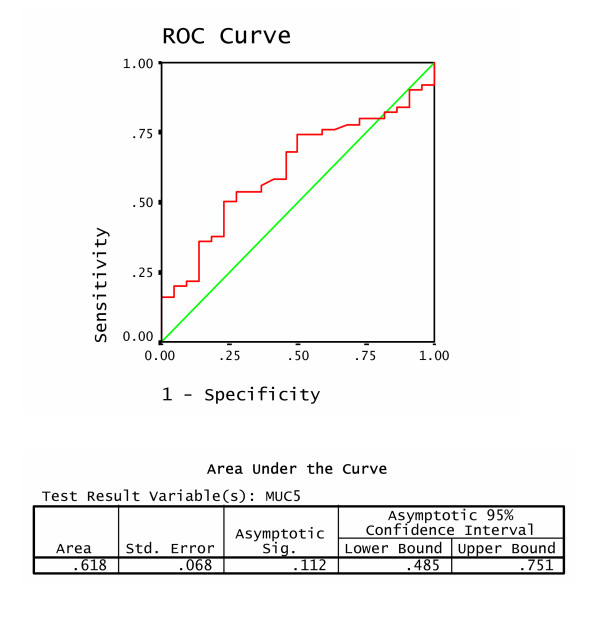
The ROC curve-comparing controls with colorectal carcinoma and polyp patients shows 0.618 of validity. ROC = receiving operating characterizing. The cut off level was taken as OD 0.225. At this value maximal sensitivity 54% and specificity 73% were attained.

In order to determine the positive cut off level of serum MUC5AC Ab, a ROC (receiving operating characterizing) curve was constructed between the normal and pathologic (polyps and colorectal carcinoma patients) groups. The cut off level was taken as OD 0.225. At this value maximal sensitivity 54% and specificity 73% were attained (Figure 1). We found that >70% of healthy controls expressed antibody levels <OD 0.225. Mean and median serum MUC5AC antibody results at a 1:20 antibody dilution is shown in Table 2. Total mean antibody levels were higher in colorectal carcinoma patients when compared to the control population (p = 0.011) and the polyp population (p = 0.049). No difference was found between polyp group and control group (p > 0.05).

**Figure 2:**
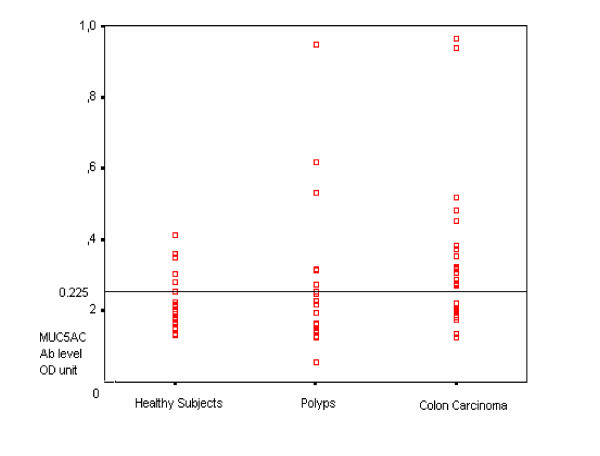
Incidence of circulating MUC5AC antibody in sera of healthy subjects, polyps, and colorectal carcinoma. As an arbitrary cut-off level 0,225 OD units was chosen. OD: Optical density.

Figure 2 shows the number of MUC5AC antibody positive individuals. MUC5AC antibody positive individuals were significantly higher in colorectal carcinoma 60% than in healthy controls 27.3% (p = 0.019). The number of positive individuals in the polyp group 45% was not significantly different from the healthy controls (p = 0.231).

The polyps that have size between 1 and 2 cm had higher incidence of serum MUC5AC Ab positivity than polyps smaller than 1 cm size (75%–40%) (p > 0.05). MUC5AC antibody positivity was seen in 66.6% of hyperplastic polyps, 40% of tubular adenoma, and 67% of tubulovillous adenoma. Incidence of MUC5AC Ab positivity was higher in patients with tubulovillous adenoma than patients with tubular adenoma (66.7%–38.5%; p > 0.05). Probably, because the number of polyps in each different polyp group were small, the differences are not statistically significant. Patients with villous adenoma didn't have MUC5AC Ab in sera and MUC5AC expression in polyp specimen. Presence of dysplasia didn't cause any difference in MUC5AC Ab positivity in sera and MUC5AC expression in polyp specimens. This might be due to a low number of polyps with dysplasia.

Of all polyp patients, 54% of group I, 33.3% of group II and 33.3% of group III had high levels of antibody. The differences between groups were not significant. We compared the presence of MUC5AC antibody with the expression of MUC5AC in the resected polyp tissue. In group I patients, 83.3% of the patients with high serum MUC5AC antibody also had MUC5AC expression in the pathology specimens (Table 3). Among antibody negative patients in group I, 80% had negative MUC5AC expression in their polyp specimen. Within Group II and III, MUC5AC expression in the pathology specimens did not correlate with serum MUC5AC antibody levels. Having MUC5AC expression at polyp specimens cause antibody formation against MUC5AC antigen at patients sera in the same time period. Having MUC5AC positive polyps at past history did not cause permanent MUC5AC antibody positivity. In polyp patients IgM antibodies were predominantly found.

### Relationship of MUC5AC antibody positivity with clinicopathologic parameters of colorectal carcinoma patients

Although these were not statistically significant, serum MUC5AC antibody positivity was higher in patients with poor prognostic parameters, these include tumors located in the colon (66.7% of MUC5AC Ab positivity in colon located tumors and 33.3% in rectum located tumors), at advanced stage (50% in Dukes B, 45.5% in Dukes C, 72.8% MUC5AC Ab positivity in Dukes D tumors), presence of preoperative metastases (72.8% vs 52.6%), and with poor and mucinous differentiated tumors (50% positivity in well differentiated tumors, 50% in moderately, 100% in poor differentiated tumors and 100% MUC5AC Ab positivity in mucinous differentiated tumors) (Table 4; p > 0.05). These parameters also negatively affect the OVS in our study groups (Table 1). IgG antibodies were predominantly found in colon carcinoma patients.

#### Disease free survival (dfs)

Average disease free survival was 14.2 months. Mean DFS was 19.91 for MUC5AC antibody negative patients and 10.38 months for MUC5AC antibody positive patients. This difference was not statistically significant (p = 0.122).

#### Overall survival (ovs)

**Figure 3:**
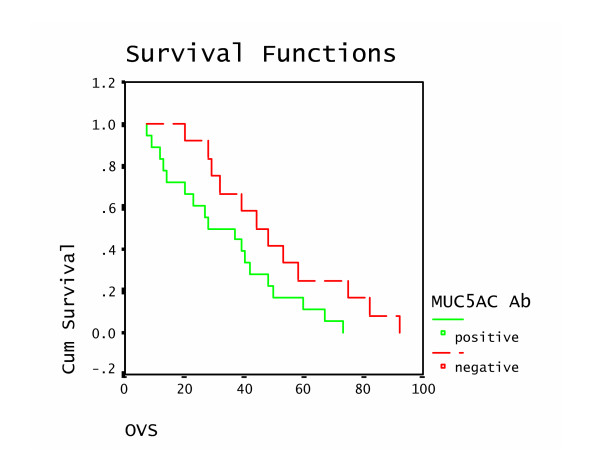
Survival curve of the colorectal tumor patients with MUC5AC antibody positive and MUC5AC antibody negative determined by the Kaplan Meier method. MUC5AC antibody positive patients showed significantly worse survival than those with MUC5AC antibody negative patients (p = 0.052). Cum Survival = Probability of cumulative survival. OVS = Overall survival.

Average of overall survival of the patients was 40.3 months (7–92 months). Mean OVS was 50 months for patients with MUC5AC antibody negative patients and 33.83 months for MUC5AC antibody positive patients (p = 0.052; Figure 3). MUC5AC antibody level did not significantly affect on prognosis in multivariate analyses.

## Discussion

In the present study we have demonstrated the presence of antibodies to MUC5AC epitopes in sera of healthy individuals, patients with polyps and colorectal carcinoma.

MUC5AC is normally expressed in respiratory, reproductive and gastric mucosa. Qualitative and quantitative alterations in the expression of the MUC5AC have been reported in both preneoplastic and neoplastic lesions. The expression decreases following neoplastic transformation in the stomach [15-18], gallbladder [19,20] and endocervix [21,22], de novo expression is seen in ovarian cancer [43], breast cancer [44], preneoplastic and neoplastic lesion of pancreas [45-48] and intrahepatic bile duct [45,49].

Genetic events that stimulate MUC5AC expression in the colon occur early in carcinogenesis. Studies have shown very low levels of MUC5AC in normal colonic mucosa [28]. MUC5AC expression increases in polyps of intermediate stage and size or with villous histology or low-grade dysplasia. It was stated that with an increasing degree of dysplasia expression decreases [25,26]. In an immunohistochemical study using non-tandem repeat antibodies, there was de novo expression of MUC5AC in 23/36 carcinomas. More MUC5AC protein was detected in well and moderately differentiated tumors than in poorly differentiated tumors (31).

In the present study, MUC5AC expression was found in 46.5% of polyp specimens. Serum MUC5AC antibody correlated well with the presence of MUC5AC expression in polyp specimens. Past history of polyps did not seem to confer permanent immunity. Only 33% of patients with history of polyps had high level of MUC5AC antibody levels. MUC5AC antibody positivity was seen in 66.6% of hyperplastic polyps, 40% of tubular adenoma, and 67% of tubulovillous adenoma. Incidence of MUC5AC Ab positivity was higher in patients with tubulovillous adenoma than patients with tubular adenoma (66.7%–38.5%; p > 0.05). In probability, because of the small numbers of polyps in each different polyp group, the differences were not statistically significant. Antibodies in polyp patients were primarily of the IgM isotype.

In our previous study [32], we demonstrated that colorectal carcinoma patients with MUC5AC negative tumors had poor clinicopathological parameters and showed worse survival than patients having MUC5AC positive tumors. The presence of MUC5AC antibodies in the serum of colorectal carcinoma patients showed no significant correlation with various clinicopathological parameters in our results. With tumor progression, immunosupression may occur during the course of disease. These patients may be unable to respond to MUC5AC on the tumor, thus antibody levels may not correlate with clinicopathologic parameters. We were unable to determine the expression of MUC5AC in the tumor tissues because we could not access these tissues in the retrospective study. Without measurements of tumor associated MUC5AC antigen and circulating immune complex concentrations, it is not possible to determine whether the decrease in circulating antibody is due to the sequestering of antibody by the tumor or in the form of immune complexes or to decreased antibody production due to immunosuppression. However, the different antibody levels in our study still could be prognostic indicators. In this retrospective study, we have found a decreased in DFS and OVS in patients who have high free circulating MUC5AC antibody.

IgG antibodies were predominantly found in colorectal carcinoma patients. A significant IgG antibody response to MUC5AC in colorectal carcinoma patients may be indicative of a CD4 helper T cell response. Patients were faced firstly with MUC5AC Ag at polyp tissues. MUC5AC antigen in polyps caused IgM type antibody formation. In adenoma-carcinoma sequence, with facing to persistent MUC5AC antigenity caused IgG type immune response. There is no data regarding the cellular immune responses to MUC5AC. Building on our previous results, we could suggest that the serum MUC5AC antibody level would be low in colorectal carcinoma patients with favorable outcome for these tumors would be expressing more MUC5AC. Free antibody could form immune complexes with cells or circulating antigen, thereby removing it from the serum. In more malignant tumors with low MUC5AC, measurable circulating free antibody levels would be high.

The technique of ELISA to detecting serum MUC5AC antibody level could be improved by examining two issues. Firstly, we used only one repeat sequence of 8 aminoacid. The sensitivity of ELISA may be increased by using at least 2 repeats of tandem repeat unit. It might provide epitopes for better recognition of antibodies present in sera. The other issue is that there are different MUC5AC glycoforms that encoded with MUC5AC gene. Also MUC5AC glycopeptides can be efficiently produced by chemoenzymatic synthesis (17). Several monoclonal antibodies were also recognized epitopes encoded by the MUC5AC gene (29). Further studies can be done by using different MUC5AC glycopeptides and monoclonal antibodies in order to better discriminate the most accurate patients to examine.

## Conclusion

In conclusion, this is an initial study on a limited number of patients showing some promising data. It shows naturally occurring antibodies reacting with the core protein of the MUC5AC mucin in the serum of healthy people, polyp and colorectal carcinoma patients. In polyp patients, the presence of serum MUC5AC antibody correlates with the expression of MUC5AC antigen in the patients polyp specimen. In colorectal carcinoma patients we demonstrate the presence of serum MUC5AC antibodies is associated with decreased survival. This may correlate with our previous finding that decreased survival is associated with low expresion of MUC5AC.

Circulating MUC5AC antigen and antibody-antigen immune complexes could be measured in order to test the sequestering of antibody. Further studies in a large number of patients may show the presence of serum MUC5AC antibody is a prognostic factor for colorectal carcinoma, and determine if a immune response has any functional antitumor activity and/or whether stimulation of this response by vaccination strategies will lead to clinically beneficial antitumor activity. The ultimate goal would be to induce antitumor activity at the early carcinogenesis or at the polyp stage to prevent the carcinoma.

## Competing interests

The author(s) declare that they have no competing interest.

## Authors' contributions

B.K participated in the design of the study, carried out the experimental studies and the preparation of the manuscript.

J.M participated in the design of the study and critically reviewed the manuscript.

A.S participated the statistical analysis and reviewed the manuscript.

## Pre-publication history

The pre-publication history for this paper can be accessed here:



## Figures and Tables

**Table 1:** Characteristics of colorectal carcinoma patients and prognostic effect on DFS and OVS.

	**Number**	**(%)**	**DFS (months)**	**P value**	**OVS (months)**	**P value**
Age						
60≤	17	56.7	10.5	**0.0184**	34	0.216
>60	13	43.3	18.9		48.4	
Sex						
Female	12	40	18.2	0.221	42.2	0.782
Male	18	60	11.5		39	
Tumor location						
Colon	24	80	10.1	**0.0272**	34.8	**0.0111**
Rectum	6	20	30.3		62	
Dukes Stage						
A	-					
B	8	26.7	25.7	**0.0002**	50.2	**0.0076**
C	11	36.7	17.8		48.4	
D	11	36.7	2.18		24.9	
Histologic type						
Adeno Ca	27	90	14.92	0.455	42.55	**0.0243**
Mucinous Ca	1	3.3	10.0		13.0	
Adenosquamous Ca	1	3.3	13.0		27.0	
Adenocarcinoid Ca	1	3.3	0.0		20.0	
Differentiation						
Well differentiated	6	20	8.3	**0.0407**	46.3	**0.002**
Moderately differentiated	19	63.3	18.6		43.9	
Poorly differentiated	4	13.3	5.5		21.7	
Colloid (mucinous)	1	3.3	0.0		9	
Metastatic Lymph Node						
Absent	8	26.7	25.7	**0.0398**	50.25	0.259
Present	22	73.3	10.0		36.68	
Preoperative Metastasis						
Absent	19	64.3	21.1	**0.000**	49	**0.0018**
Present	11	36.6	2.18		24.91	
Serum MUC5AC Ab level						
Negative	12	40	19.91	0.153	50.0	**0.052**
Positive	18	60	10.38		33.83	

DFS = Disease Free Survival

OVS = Overall Survival

**Table 2:** MUC5AC serum antibody levels in healthy controls, in polyp and malignant colorectal carcinoma patients. Serum mean MUC5AC antibody levels were higher in colorectal carcinoma patients when compared with polyp patients (p = 0.049) and a control population (p = 0.011).

	Healthy controls (n = 22)	Polyp group (n = 20)	Carcinoma group (n = 30)
* No. > 0,225	6 (27.3%)	9 (45%)	18 (69%)
Mean	0.219 OD	0.268 OD	0.322 OD
Median Range (min-max)	0.195 OD (0.13–0.41)	0.207 OD (0.06–0.95)	0.277 OD (0.12–0.96)

* Number of patients that have higher MUC5AC antibody level than 0.225 OD.

OD = Optical density

**Table 3:** Serum MUC5AC antibody levels (OD unit) and MUC5AC expression in pathology specimens of polyp patients.

	**Group I (n = 11)**		**Group II (n = 6) and Group III (n = 3)**		
	**Serum Antibody Level**	**Expression of MUC5AC in polyp specimens**	**Serum Antibody level**	**Expression of MUC5AC in polyp specimens**	
				
				**New**	**Previous**

**Antibody positive**	**0.948**	(+)*	**0.225**	**(+)(+)**	(-)**(+)**(-)(-)
	**0.312**	**(+)**			(-)**(+)**
	**0.616**	**(+)(+)**			
	**0.317**	(-)	**0.246**	(-)	**(+)**
	**0.275**	**(+)**(-)			
	**0.227**	**(+)**	**0.53**		**(-)**

**Antibody negative**	0.165	(-)(-)	0.055	(-)**(+)**	**(+)**
	0.143	(-)**(+)**	0.163	**(+)**(-)	**(+)**
	0.219	(-)	0.195	(-)	(-)
	0.146	(-)	0.151	**(+)**	(-)(-)(-)(-)**(+)**
	0.127	(-)			
			0.14		**(+)(+)**
			0.128		(-)

*: Each parenthesis represents the histology of one polyp specimens and MUC5AC expression.

(-): No MUC5AC staining at polyp specimens

(+): MUC5AC staining at polyp specimens

HP: Hyperplastic polyp

TA: Tubuler adenoma

TV: Tubulovillous adenoma

V: Villous adenoma

OD = Optical density

**Table 4:** Serum MU5AC positivity in each clinicopathologic parameters

	**MUC5AC Ab negative**		**MUC5AC Ab positive**		
	**N**	**(%)**	**N**	**(%)**	

Age					
≤60	(5)	29.4	(12)	70.6	
>60	(7)	53.8	(6)	46.2	
					(p = 0.176)
Sex					
Female	(8)	66.7	(4)	33.3	
Male	(4)	22.2	(14)	77.8	
					(p = 0.015)
Tumor Location					
Colon	(8)	33.3	(16)	66.7	
Rectum	(4)	66.7	(2)	33.3	
					(p = 0.116)
Duke's Stage					
B	(4)	50.0	(4)	50.0	
C	(6)	54.5	(5)	45.5	
D	(27)	31.3	(8)	72.7	
					(p = 0.25)
Histologic type					
Adeno Ca	(12)	44.4	(15)	55.6	
Mucinous Ca	-	-	(1)	100	
Adenosquamous Ca	-	-	(1)	100	
Adenocarcinoid Ca	-	-	(1)	100	
					(p = 0.528)
Differentiation					
Well	(3)	50.0	(3)	50.0	
Moderate	(9)	47.4	(10)	52.6	
Poor	-	-	(4)	100	
Mucinous	-	-	(1)	100	
					(p = 0.260)
Metastatic Lymph Node					
Absent	(3)	37.5	(5)	62.5	
Present	(9)	40.9	(3)	59.1	
					(p = 0.866)
Preoperative Metastasis					
Absent	(9)	47.4	(10)	52.6	
Present	(27)	31.3	(8)	72.7	
					(p = 0.219)
